# Optimizing Superhydrophobic Coatings: The Role of Catalysts, Additives, and Composition on UV and Thermal Stability of Inverse Vulcanization Polymers

**DOI:** 10.1021/acsapm.4c02634

**Published:** 2025-01-06

**Authors:** Vinicius Diniz, Susanne Rath, Colin R. Crick

**Affiliations:** † School of Engineering and Materials Sciences, Queen Mary University of London, London, E1 4NS, U.K.; ‡ Institute of Chemistry, University of Campinas, 13083-970 Campinas, Brazil

**Keywords:** Inverse vulcanization, Mechanical durability, Sulfur, Superhydrophobic, Water repellence

## Abstract

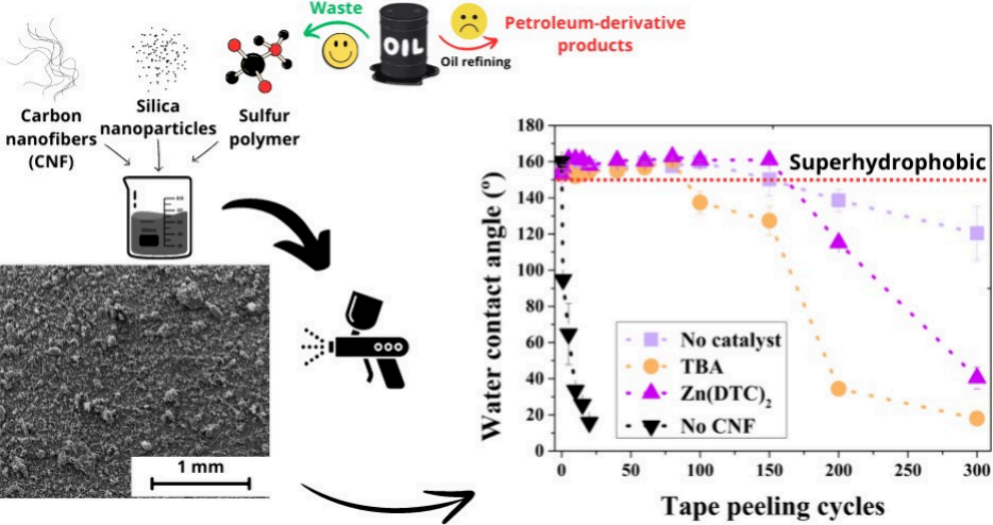

Inverse vulcanization (IV) enables the production of sustainable polymer from sulfur waste, offering hydrophobic, fluorine-free, and superhydrophobic coatings. However, these materials need adhesion improvements for enhanced durability. This study has developed an epoxy-, fluorine-, and metal-free superhydrophobic coating using the spray-coating of carbon nanofibers (CNFs), silica nanoparticles, and IV polymers on glass. An optimized formula of 28% sulfur, 20 mg/mL CNFs, 25 mg/mL silica, and 80 mg/mL polymer–was established. Zn(DTC)_2_-catalyzed coatings retained superhydrophobicity for 150 tape peeling cycles, up to 250 °C, and 6 h of UV–C exposure, demonstrating a straightforward, eco-friendly approach to durable, versatile superhydrophobic coatings.

Superhydrophobic materials are valued for self-cleaning, water purification, antibacterial activity, and drag-reduction applications.^1^ Despite their potentially environmentally benign fabrication processes, their composition often includes persistent chemicals (e.g., fluorinated macromolecules), petroleum-derived polymers–particularly of concern in the end-of-life disposal of these materials, and metal alloys.^2−4^ “Inverse vulcanization” copolymerization offers a sustainable way to repurpose sulfur waste, showcasing its versatility across various research areas.^5−12^ Inverse vulcanization polymers retain notable properties of elemental sulfur, including photocatalytic, antibacterial, and electrochemical activities, while being synthesized through a solventless, scalable, and environmentally friendly method.^13,14^ To date, limited studies have explored the use of inverse vulcanization polymers in the fabrication of superhydrophobic materials, showcasing interesting properties (e.g., self-cleaning, anticorrosion, and antibacterial properties) but with limited mechanical durability.^15,16^ Using tape peeling tests, Miao et al.^17,18^ reported sulfur polymer coatings with the highest durability so far, although this solution required the application of epoxy, fluorinated compounds, and metal derivates.

Recent advancements in catalytic inverse vulcanization copolymerization have shown the advantage of lowering copolymerization temperature requirements while synthesizing polymers with diverse chemical and mechanical properties.^19^ Nonthermal routes have also be proposing for synthesizing inverse vulcanization sulfur polymer;^20,21^ however this study will focus on understanding the effects of catalysts on the resulting polymers. Since first being reported, various catalysts have been utilized, with zinc(II) diethyldithiocarbamate (Zn(DTC)_2_) demonstrating the most promising results, reducing the reaction time and H_2_S generation.^19,22^ More recently, trialkyl amines were also proposed as alternative catalysts to synthesize inverse vulcanization polymers.^23^ Despite the progress in alternative synthetic pathways, a gap remains in understanding how the presence of catalysts influences the physical and chemical properties of the polymers and thus their applicability.

In this study, we aimed to fabricate a fluorine-, epoxy-, and metal-free superhydrophobic coating by spray coating a layer comprising carbon nanofibers (CNFs)/Silica/inverse vulcanization sulfur polymers [both catalyzed (Zn(DTC)_2_ or tributylamine (TBA)) and uncatalyzed] onto glass substrates. The addition of CNFs and silica was required to facilitate the creation of a hierarchical roughness structure necessary for achieving superhydrophobicity while also enhancing mechanical durability.^24−26^ The inverse vulcanization polymers thus acted as binder materials for the coating while providing the required hydrophobic chemistry to the coatings. Our results indicate that the addition of catalysts can influence the mechanical durability of the coatings. Optimizing the formulation resulted in a coating able to maintain superhydrophobicity after 150 tape peeling cycles, up to 250 °C, and 6 h of UV–C irradiation exposure, demonstrating outstanding mechanical robustness without any fluorine compounds or epoxy. The simple and environmentally friendly preparation and outstanding mechanical robustness make these coatings the preferred choice for practical applications.

Sulfur polymers [here synthesized using 1,3-diisopropenylbenzene (DIB)^27^] possess photocatalytic properties and can be tailored to achieve hydrophobicity by selecting hydrophobic cross-linkers, offering promising fluorine- and metal-free alternatives for superhydrophobic coatings. However, this photocatalytic behavior can also induce degradation of the polymer coating, necessitating a careful evaluation of its long-term stability and performance in practical applications.^15^ In addition, their mechanical durability is limited without the addition of adhesive compounds like epoxy.^15^ This mechanical durability is provided here by CNFs, which were added to the coating formulation and embedded in the coating material. To help understand the effect of formulation components (including the concentration of CNFs, silica, poly(S-DIB-TBA), and sulfur amount) on the water contact angle (WCA) and tape peeling durability, an experimental design approach (Central Composite Design, CCD) was employed (see Supporting Information for further detail, Table S1). To facilitate the results presentation, the coatings were termed as _X_CNF-_Y_SiO_2_@_Z_poly(_a_S-DIB-A), where *a*, *x*, *y*, and *z* are the content of sulfur, the concentration (mg/mL) of CNF, silica and inverse vulcanization polymer in the coating formulation, respectively, and A is the catalyst (Zn(DTC)_2_ or TBA), if any. For instance, a coating containing 10 mg/mL of CNF, 50 mg/mL of SiO_2_, 50% of sulfur, 100 mg/mL of polymer, and TBA as catalyst is denoted as _10_CNF-_50_SiO_2_@_100_poly(_50_S-DIB-TBA)._._

Initially, the poly(S-DIB-TBA) series were synthesized and characterized by FTIR (Figure S1), NMR (Figures S2–S7), PXRD (Figure S8), and DSC (Figure S9). Characterization of the materials revealed that the polymers exhibit similar FTIR, NMR, and PXRD spectra. However, a higher sulfur content was found to correlate with a lower glass transition temperature (*T*_g_) (Figure S9), which is attributed to the increased length of sulfur–sulfur chains in the polymers.^28^ Then, 18 coating formulations (Table S2) were prepared and spray-coated onto microscope slides (Figure 1A). Seventeen out of 18 coatings showed WCA > 150°, except for _10_CNF-_0_SiO_2_@_55_poly(_50_S-DIB-TBA) (Figure 2). As seen in Figure 1B, coatings containing even low amounts of silica provided a rough surface, while the coatings containing no silica had limited roughness, which reduced the WCA; the addition of hydrophobic silica enhances both surface roughness and the WCA due to its chemical nature, contributing to improved hydrophobicity. Scanning electron microscopy (SEM) was principally used to examine surface roughness, which reveals a dual-scale roughness (micro/nanoscale, Figure 1B). Atomic force microscopy (AFM) was used to analyze solely the nanoscale roughness, as the microscale roughness was too high in magnitude to be measured by the AFM instrument used. Combined, these data confirmed that the superhydrophobic characteristics of the coatings are derived from both surface roughness and hydrophobic chemistry. The microscale features visible via SEM (Ø ∼ 3–10 μm) were combined with nanoscale roughness (RMS roughness of 89.8 nm and average roughness of 73.3 nm measured by AFM, Figure S10 and Table S3). Although showing a WCA > 150°, _10_CNF-_50_SiO_2_@_100_poly(_50_S-DIB-TBA) presented a surface sticky to water, characterized by a rolling angle (RA) of 35°, while the other coatings showed very low RAs (<3°). This could be explained by a high concentration (100 mg/mL) of poly(S-DIB-TBA) in the formulation with an intermediate level of silica nanoparticles, resulting in the polymer filling in the gaps within the roughness of the coating (Figure 1B).

**Figure 1:**
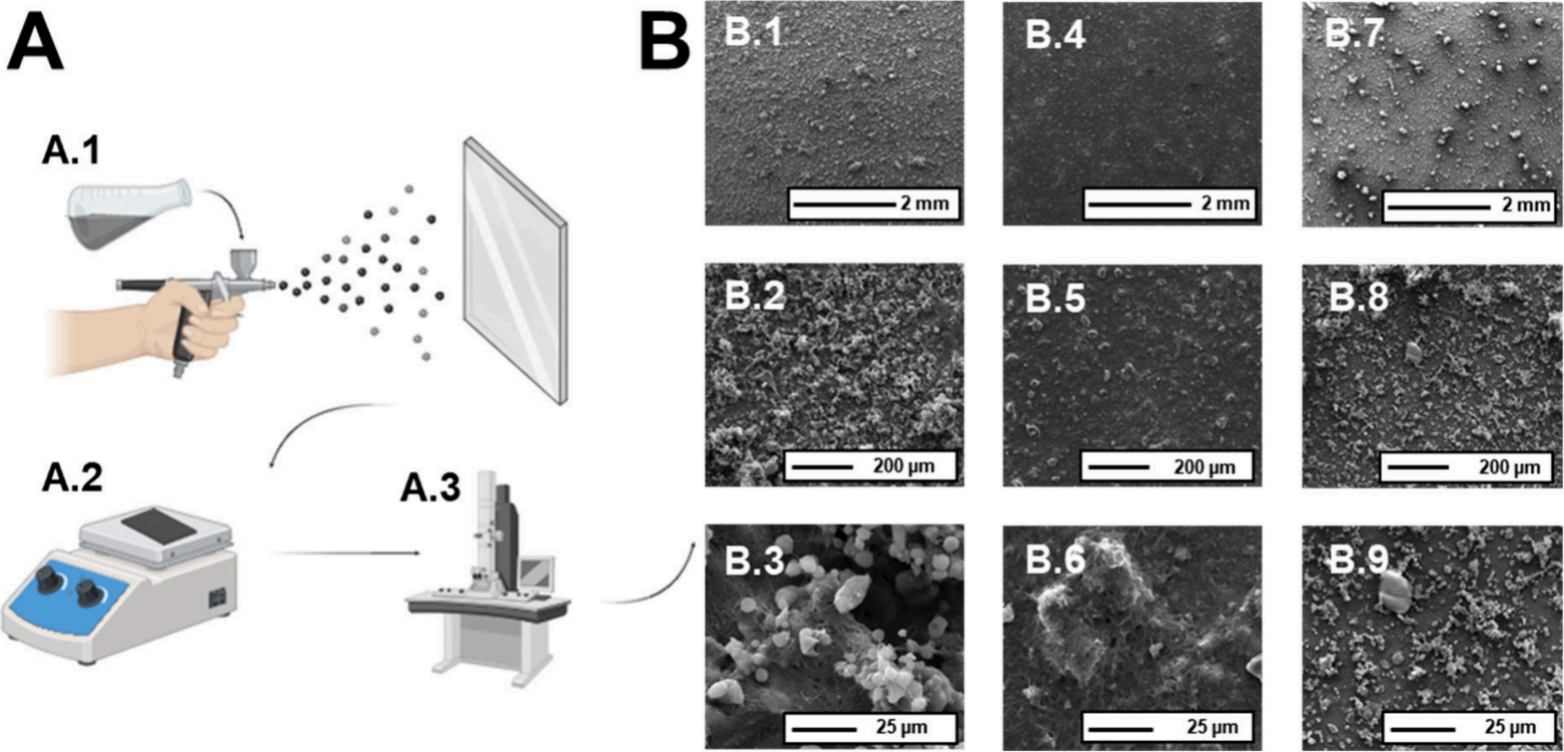
(A) Schematic of coating on substrate: (A.1) – coating formulation deposited onto glass slides by spray coating; (A.2) coating dried at 80 °C; (A.3) morphology characterization by scanning electron microscopy. (B) Top-down SEM micrographs showing the morphology of (B.1–B.3) _15_CNF-_75_SiO_2_@_77.5_poly(_28_S-DIB-TBA), (B.4–B.6) _10_CNF-_0_SiO_2_@_55_poly(_50_S-DIB-TBA), and (B.7–B.9) _10_CNF-_50_SiO_2_@_100_poly(_50_S-DIB-TBA)_._.

**Figure 2:**
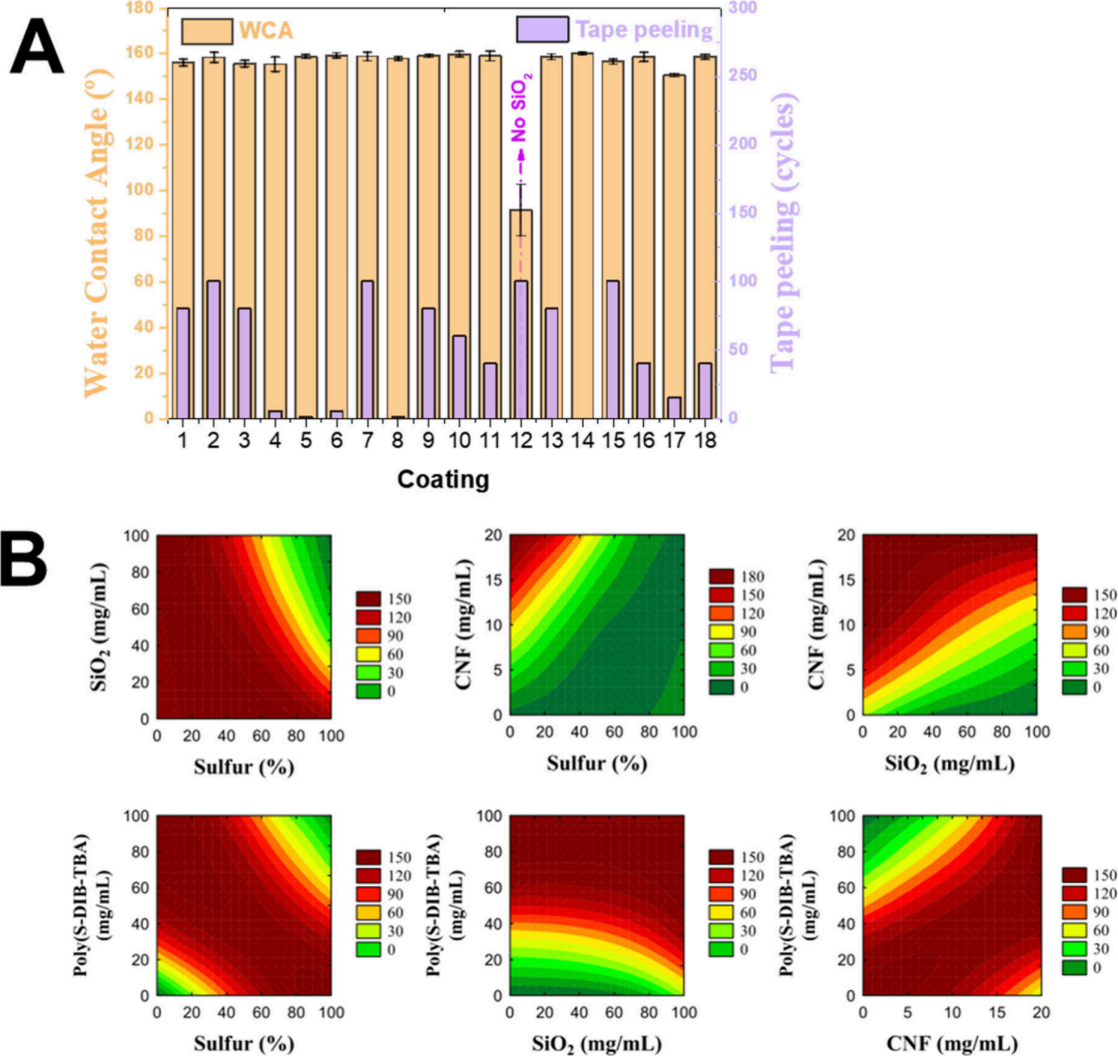
(A) WCA and tape peeling durability of various coating formulations. Tape peeling durability is represented by the number of cycles required for the water contact angle to drop below 150°, except for coating 12 (no silica), where a decrease of 10% in the WCA was considered. (B) Contour diagrams showing the binary interactions of coating formulation components in the durability of tape peeling.

It was found that all parameters (except for the concentration of silica) impacted the tape peeling data (Figure 2B). As expected, the mechanical durability of the coatings was significantly influenced by the presence of CNFs, with a higher concentration leading to a higher durability. An optimal concentration of poly(S-DIB-TBA) was identified at 80 mg/mL (Figure 2B). The sulfur content in the polymer also impacted the mechanical durability, with a lower sulfur content leading to increased durability. As observed in Figure S9 and corroborated by scientific literature,^28^ polymers with lower sulfur content demonstrated higher *T*_g_ than those with higher sulfur content. This suggests that *T*_g_ plays a significant role in the mechanical durability of the coatings. The sulfur content and the material’s physical properties could also impact the mechanism in which the coating is damaged,^29^ therefore affecting the WCAs measured.

The optimal coating formulation (28% sulfur, 20 mg/mL CNFs, 25 mg/mL silica, and 80 mg/mL poly(S-DIB-TBA)) was used to further investigate the influence of catalysts on the mechanical durability of the coatings. For this, poly(S-DIB), poly(S-DIB-TBA), and poly(S-DIB- Zn(DTC)_2_) were synthesized and characterized (see Supporting Information, Figures S11–S16). While the additives have improved the WCA, the catalyst did not significantly affect the wettability (WCA/RA) whereby all fabricated coatings (*p* > 0.05) showed superhydrophobic characteristics (Figure 3A). Conversely, it was clear that the presence of a catalyst and/or catalyst type influenced the mechanical durability of the coatings (Figure 3B and 3C). Whereby, catalyzed reactions generally fabricated higher *T*_g_ polymers, which in turn showed higher durability for tape peeling (*p* < 0.05) and table abraser tests (*p* < 0.05) with a 1.5-fold higher durability. Additionally, it was observed that the catalyst affects the UV–C (λ = 254 nm) stability of the coatings with catalyzed polymers showing longer stability (Figure 3D). Coatings also showed remarkable thermal stability, with both catalyzed ones maintaining their superhydrophobic properties even at 250 °C (Figure 3E). To demonstrate this, EDS mapping was used to track the sulfur content of the coatings after heating at 250 °C for 1 h (Figure 3F). The amount of sulfur was shown to differ between the polymers, with the poly(S-DIB) showing 14.3% sulfur, while poly(S-DIB-TBA) and poly(S-DIB-Zn(DTC)_2_) showed 17.9% and 26.9%, respectively. These data corroborate the TGA data, as there was a greater mass loss in poly(S-DIB) at 250 °C than in other polymers. Sulfur is likely lost when there is a generation of oxidized sulfur gaseous species, which is prevented to some degree when the reaction is catalyzed. This is caused by a difference in the chemical structure of the polymers, which is reflected in the differing thermophysical properties (e.g., *T*_g_).

**Figure 3:**
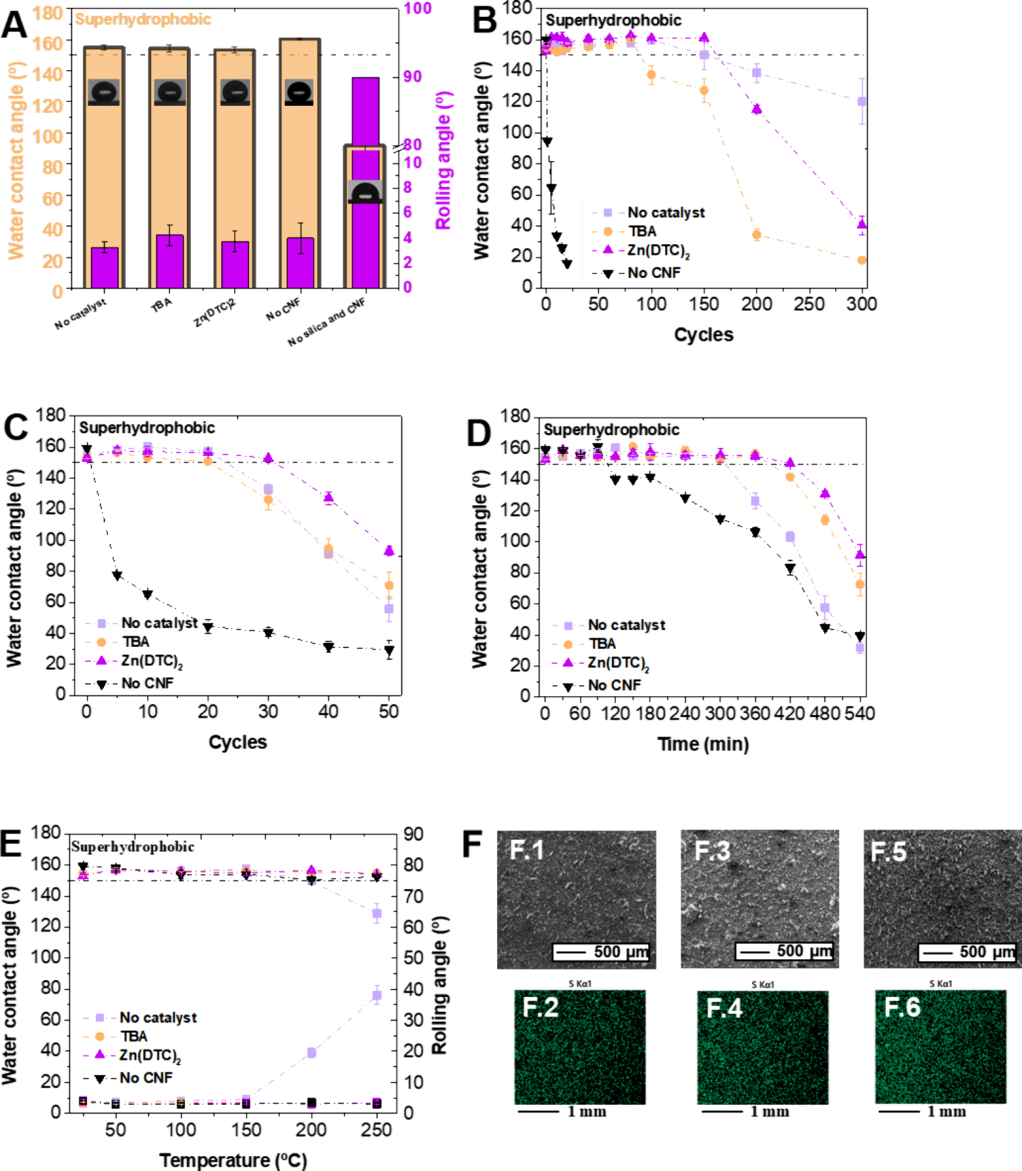
Effect of catalyst (TBA and Zn(DTC)_2_) and CNF on (A) WCA and rolling angle, (B) tape peeling durability, (C) taber abraser durability, (D) UV stability, and (E) thermal stability of the coatings. No CNF (only silica was used as a roughness agent) coating was fabricated with poly(_28_S-DIB-TBA). (F) SEM micrographs and EDS map of sulfur of (F.1 and F.2) _20_CNF-_25_SiO_2_@_80_poly(_28_S-DIB), (F.3 and F.4) _20_CNF-_25_SiO_2_@_80_poly(_28_S-DIB-TBA), and (F.5 and F.6) _20_CNF-_25_SiO_2_@_80_poly(_28_S-DIB-Zn(DTC)_2_).

To date, little is known about the effect of catalysts on the applicability of inverse vulcanization sulfur polymers, despite reports indicating that catalyzed copolymerization reduces the generation of H_2_S gas, requires shorter vitrification times, and yields higher reaction efficiencies.^19,22^ Additionally, catalysts can also alter the distribution of sulfur within the polymers, which may increase or decrease the cross-linking degree, affecting the *T*_g_ and, consequently, the mechanical properties of the polymers.^19^ Here, we observed that while Zn(DTC)_2_ increased *T*_g_, TBA led to lower values, directly impacting the properties of the coatings. These findings suggest that, in addition to adding CNF to the coatings, modifying copolymerization by introducing catalysts can further tailor the properties of the coatings to desired specifications.

In conclusion, this study successfully fabricated epoxy-, fluorine-, and metal-free superhydrophobic coatings by combining CNFs, silica, and inverse vulcanization sulfur polymers. Optimization of the coating formulation components via experimental methods resulted in a polymer with 28% sulfur content, alongside 20 mg/mL CNFs, 25 mg/mL silica, and 80 mg/mL polymer. The coatings exhibited remarkable mechanical durability, surpassing previous literature benchmarks.^15,16^ Furthermore, the mechanical durability of the coatings was enhanced by adding the Zn(DTC)_2_ catalyst during polymer synthesis, leading to the production of polymers with higher *T_g_*. Comparative analysis revealed that poly(S-DIB-Zn(DTC)_2_) coatings displayed superior mechanical durability as well as UV and thermal stability compared to uncatalyzed or TBA-catalyzed reactions. Overall, this study demonstrates a straightforward and environmentally friendly approach for producing robust superhydrophobic coatings that can be replicated across various settings. For future studies, we recommend a detailed investigation into the effects of catalysts on polymer structure, as this may provide deeper insights into their impact on the final properties of the polymers.

## Experimental Methods

Inverse vulcanization polymers were synthesized by inverse vulcanization using DIB as cross-linker.^28^ The polymers were synthesized with or without the addition of catalysts (Zn(DTC)_2_ or TBA). Briefly, elemental sulfur (0.6–9.4 g), DIB (0.1–8.9 mL), and TBA (catalyst) (0.5 mL) were vortexed for 60 s and sonicated for 30 min for complete homogenization. The mixture was then stirred at 140 °C until a dark color appears (approximately 25 min) before being transferred to a silicon mold for curing at 140 °C for an additional 18 h. For the poly(S-DIB) catalyzed with Zn(DTC)_2_ the same procedure was followed except that 3% w/w of catalyst was used instead of 5% w/w. For the uncatalyzed polymer, no catalyst was added, and the mixture was stirred at 160 °C instead of 140 °C.

To achieve a homogeneous dispersion, CNFs, hydrophobic-silica nanoparticles (prepared as described by Upton et al.^15^), inverse vulcanization polymer, and chloroform were vortexed for 60 s and sonicated for 15 min. The resulting mixture was then sprayed using a compression pump and airbrush gun (Voilamart) (pressure set as 2 bar) onto a microscope slide [76 × 26 mm; thickness: 1.0–1.2 mm, Academy-science (UK)] and dried at 80 °C for 30 min (see Figure 1). The airbrush was used at a distance of ∼4 cm from the substrate, and the coatings were sprayed manually, applying a volume of 3 mL with a back-and-forth motion for approximately 30 s.

The wettability, morphology, and durability performances of the coating were measured. Further details regarding these measurements and polymer characterization can be found in the Supporting Information (S5).

## Supporting Information Available

Additional figures and tables providing details on the experimental design and polymer characterization, along with comprehensive descriptions of the experimental procedures (PDF)
