# Supplementary material for: Optimizing Superhydrophobic Coatings: The Role of Catalysts, Additives, and Composition on UV and Thermal Stability of Inverse Vulcanization Polymers
Source: ACS Appl Polym Mater. 2025 Jan 6;7(2):567–72. doi: 10.1021/acsapm.4c02634 (PMC11773412; doi:10.1021/acsapm.4c02634)
Supplement: Supplementary file 1 — ap4c02634_si_001.pdf [file ap4c02634_si_001.pdf]

Supporting information: Optimizing Superhydrophobic Coatings: The Role of Catalysts, Additives, and Composition on UV and thermal stability of Inverse Vulcanization Polymers

Vinicius Diniz[1,2*], Susanne Rath[2] and Colin R. Crick[1*]

[1]School of Engineering and Materials Sciences, Queen Mary University of London, London, E1 4NS, UK.

[2]Institute of Chemistry, University of Campinas, 13083-970 Campinas, Brazil.

*Authors for correspondence: c.crick@qmul.ac.uk and *viniciusdiniz994@gmail.com*

S1. Experimental Design

The CCD was systematically delineated by defining actual levels and coding levels for each parameter. High levels are denoted as +1, central levels as 0, and low levels as -1. A comprehensive representation of these actual and coded levels is presented in Table S1. Table S2 presents the details of each coating formulation alongside the corresponding results for WCA and mechanical durability.

Table S1: Actual levels and coded reaction parameters.

Factors	Code and level		
	-1	0	+1
A-Sulfur (%, w/w)[a]	28	50	72
B-CNF (mg/mL)	5	10	15
C – SiO_2 (mg/mL)	25	50	75
D-Poly(S-DIB-TBA) (mg/mL)	32.5	55	77.5

[a]Sulfur content in the poly(S-DIB-TBA). Note that 0% would lead to liquid non-polymer formulation.

Table S2: Experimental design matrix with uncoded surfaces.

Coating	Sulfur (%, w/w)	CNF (mg/mL) (mg/cm^2)^b	SiO_2 (mg/mL) (mg/cm^2)^b	Poly(S-DIB-TBA) (mg/mL) (mg/cm^2)^b	WCA (SD)^c (º)	RA (º)	Durability (tape peeling cycles)
1	72	15 (2.30)^b	75 (11.40)^b	32.5 (4.90)^b	156.1 (1.4)^c	<3	80
2	72	5 (0.75)^b	75 (11.40)^b	32.5 (4.90)^b	158.4 (2.3)^c	<3	100
3	72	15 (2.35)^b	25 (3.80)^b	77.5 (11.75)^b	155.7 (1.4)^c	<3	80
4	28	5 (0.75)^b	75 (11.40)^b	77.5 (11.75)^b	155.3 (3.1)^c	<3	5
5	72	5 (0.75)^b	25 (3.80)^b	77.5 (11.75)^b	158.7 (0.9)^c	<3	1
6	28	15 (2.30)^b	25 (3.80)^b	32.5 (4.90)^b	159.2 (1.2)^c	<3	5
7	28	15 (2.30)^b	75 (11.40)^b	77.5 (11.75)^b	158.8 (2.0)^c	<3	100
8	28	5 (0.75)^b	25 (3.80)^b	32.5 (4.90)^b	157.8 (0.7)^c	<3	1
9	50	10 (1.50)^b	50 (7.60)^b	55.0 (8.35)^b	159.1 (0.7)^c	<3	80
10	6	10 (1.50)^b	50 (7.60)^b	55.0 (8.35)^b	159.8 (1.2)^c	<3	60
11	94	10 (1.50)^b	50 (7.60)^b	55.0 (8.35)^b	159.0 (2.2)^c	<3	40
12	50	10 (1.50)^b	0 (0.00)^b	55.0 (8.35)^b	91.3 (11.3)^c	>90	100
13	50	10 (1.50)^b	100 (15.20)^b	55.0 (8.35)^b	158.6 (1.2)^c	35	80
14	50	0 (0.00)^b	50 (7.60)^b	55.0 (8.35)^b	160.1 (0.6)^c	<3	0
15	50	20 (3.00)^b	50 (7.60)^b	55.0 (8.35)^b	156.5 (1.2)^c	<3	100
16	50	10 (1.50)^b	50 (7.60)^b	10 (1.50)^b	158.6 (2.1)^c	<3	40
17	50	10 (1.50)^b	50 (7.60)^b	100 (15.20)^b	150.5 (0.6)^c	<3	15
18	50	10 (1.50)^b	50 (7.60)^b	55.0 (8.35)^b	158.6 (1.1)^c	<3	40

^{a}Sulfur content in the poly(S-DIB-TBA); ^{b}Mass per area; ^{c}Standard deviation.

S2. Poly(S-DIB-TBA) characterization.

S2.1 FTIR

The inverse vulcanization copolymerization can be track by the consumption of $-CH_2$ in the DIB molecule and the generation of C-S bonds in the poly(S-DIB-TBA) structure, which can be confirmed by the disappearance of the 886 cm^{-1} band and the appearance of the 695 cm^{-1} band, respectively (Figure S1). It is worth noting that the polymer containing 6% sulfur still had some unreacted DIB in its structure, which can be observed by the residual 886 cm^{-1} band in the FTIR spectra.

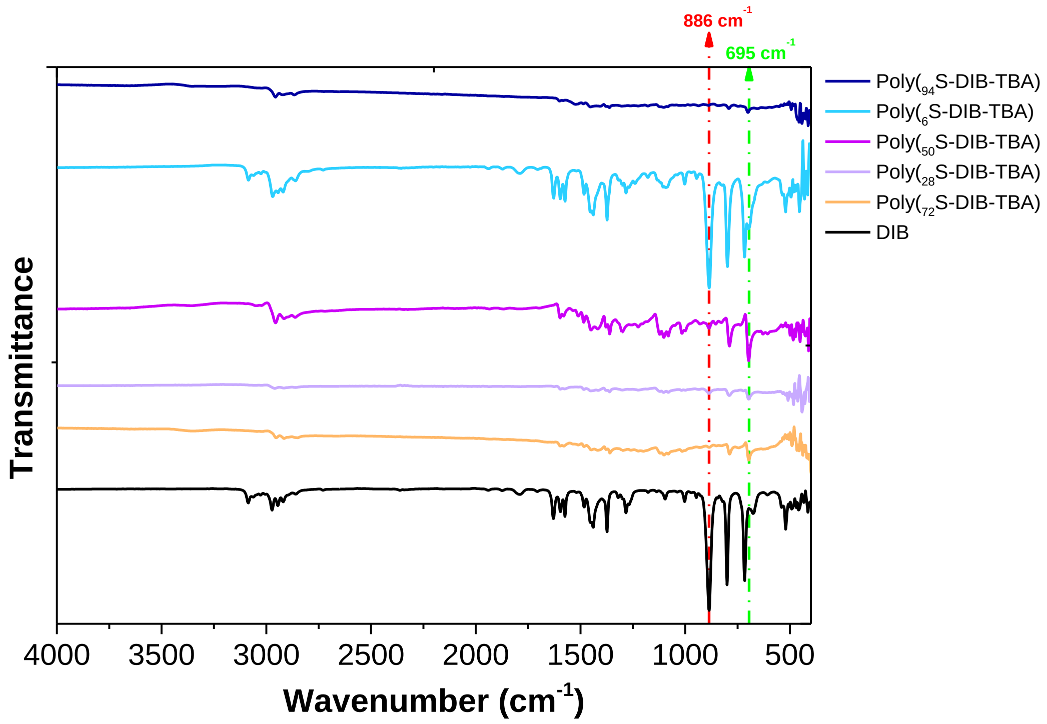

Figure S1: FTIR spectra of the poly(S-DIB-TBA) series.

S2.2 NMR

^{1}H-NMR was used to monito the polymerization and unsure successful formation of poly(S-DIB-TBA) polymers (Figure S2 – S6). The consumption of DIB can be tracked by the disappearance of methylene proton signals (δ = 5.10–5.40 ppm) (Figure S2). On the other hand, the primary framework of the copolymer poly(S-DIB-TBA) mainly comprises thiocumyl fragments, which act as the fundamental building blocks. These are complemented by minor segments containing thiopropyl or bis-thiopropyl fragments. Additionally, the end groups of poly(S-DIB-TBA) are likely either −SSH sulfanes or exposed isopropenyl groups.

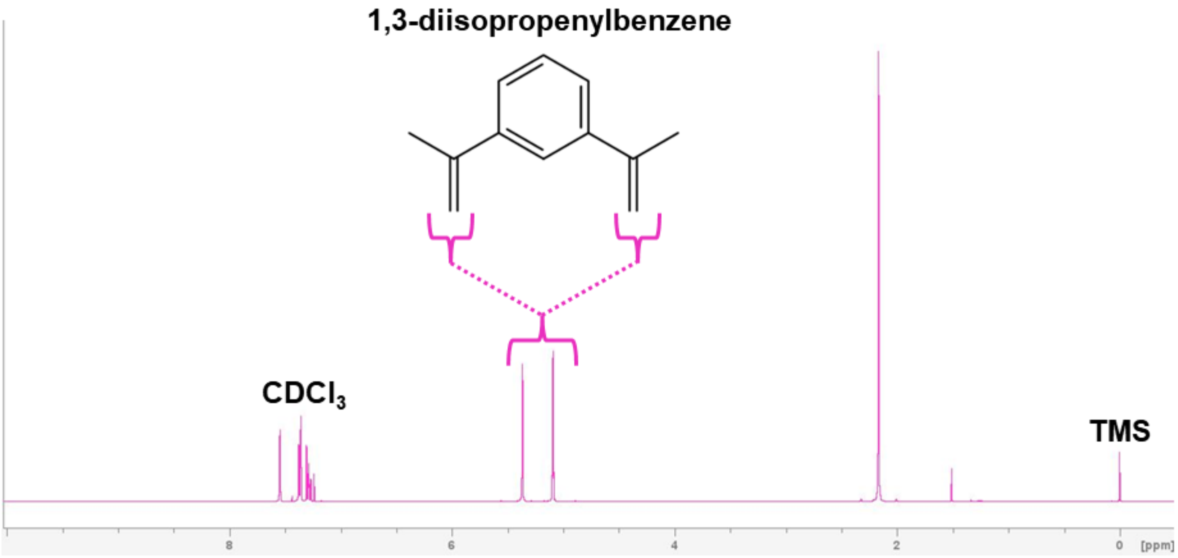

Figure S2: ^{1}H-NMR spectrum of 1,3-diisopropenylbenze.

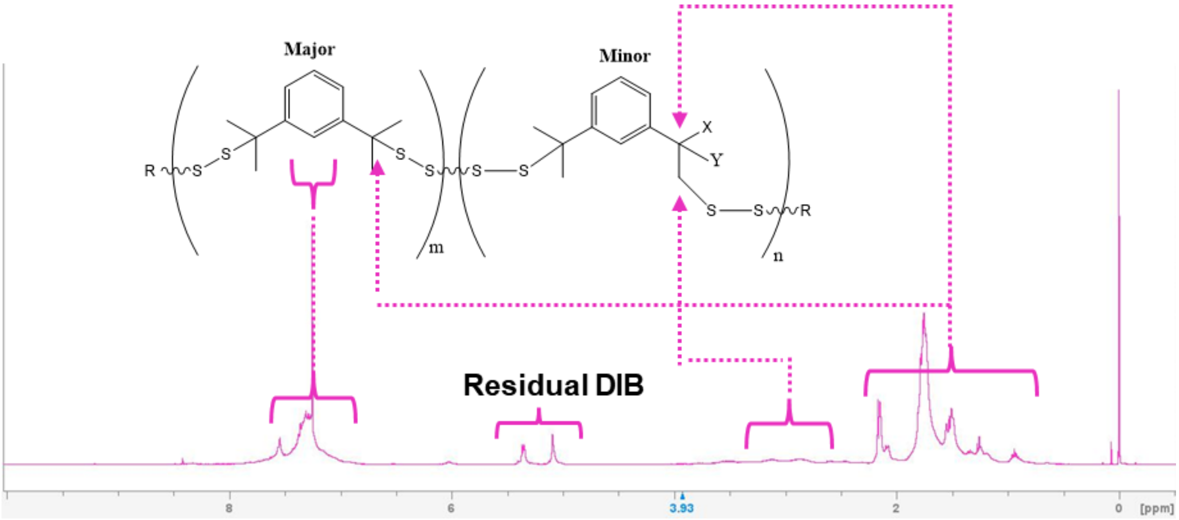

Figure S3: ^{1}H-NMR spectrum of poly($_{28}$S-DIB-TBA).

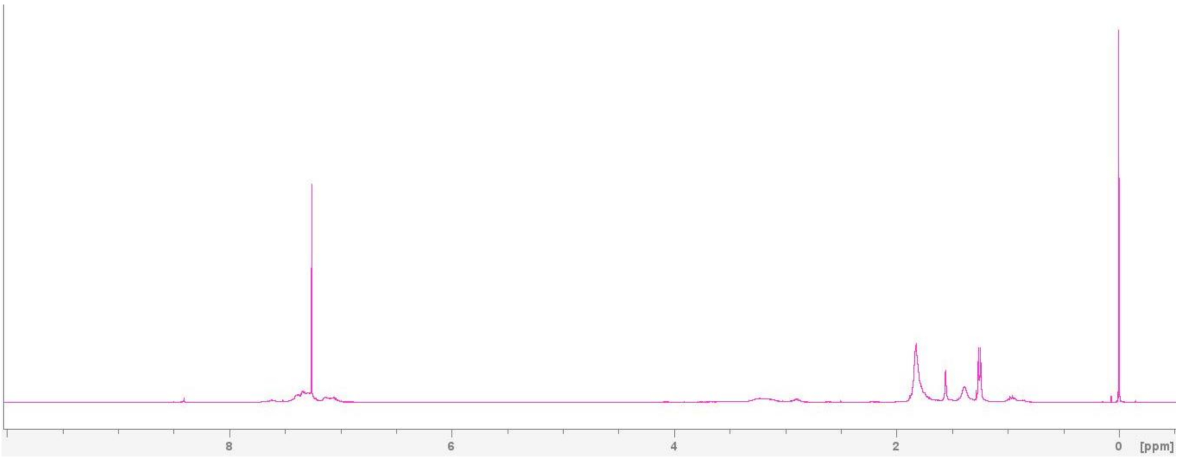

Figure S4: ^{1}H-NMR spectrum of poly($_{72}$S-DIB-TBA).

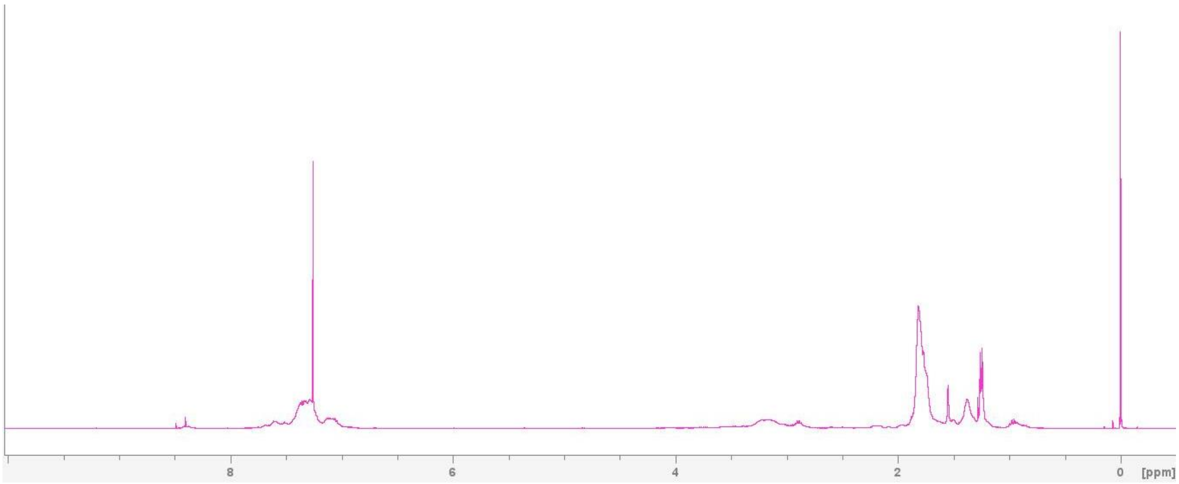

Figure S5: ^{1}H-NMR spectrum of poly($_{50}$S-DIB-TBA).

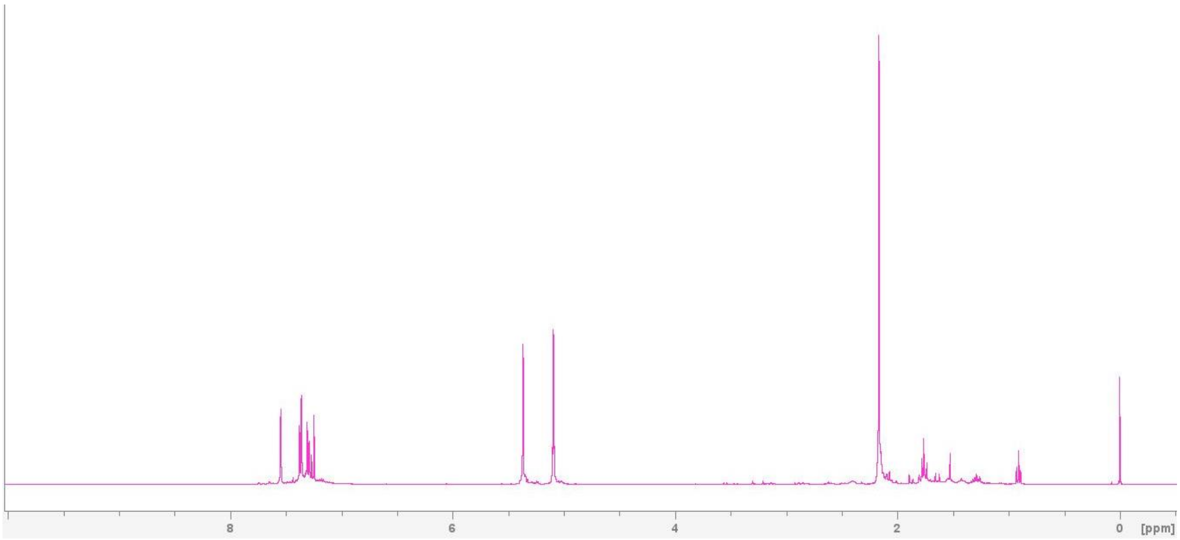

Figure S6: ^{1}H-NMR spectrum of poly($_{6}$S-DIB-TBA).

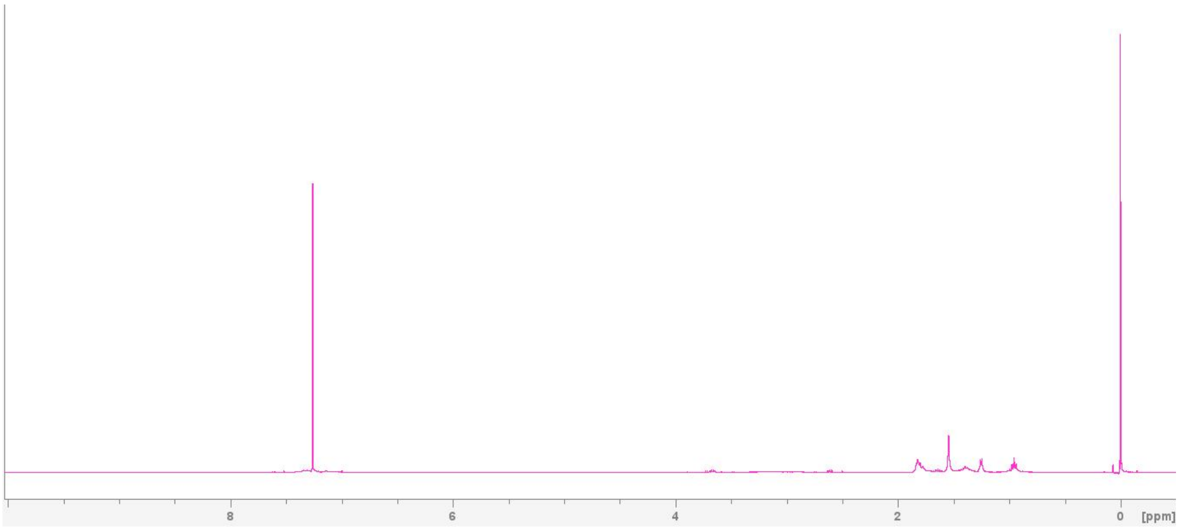

Figure S7: ^{1}H-NMR spectrum of poly($_{94}$S-DIB-TBA).

S2.3 PXRD

Sulfur consumption can be monitored by PXRD. Characteristic diffraction peaks appeared at 2θ = 16° 23°, 27°, 28°, and 32° (Figure S7). In the poly(S-DIB-TBA) series, the polymers that contained 72% and 94% sulfur had unreacted sulfur in their structure. It is worth noting that the poly($_6$S-DIB-TBA) was not analyzed by PXRD because it was in a viscous liquid form.

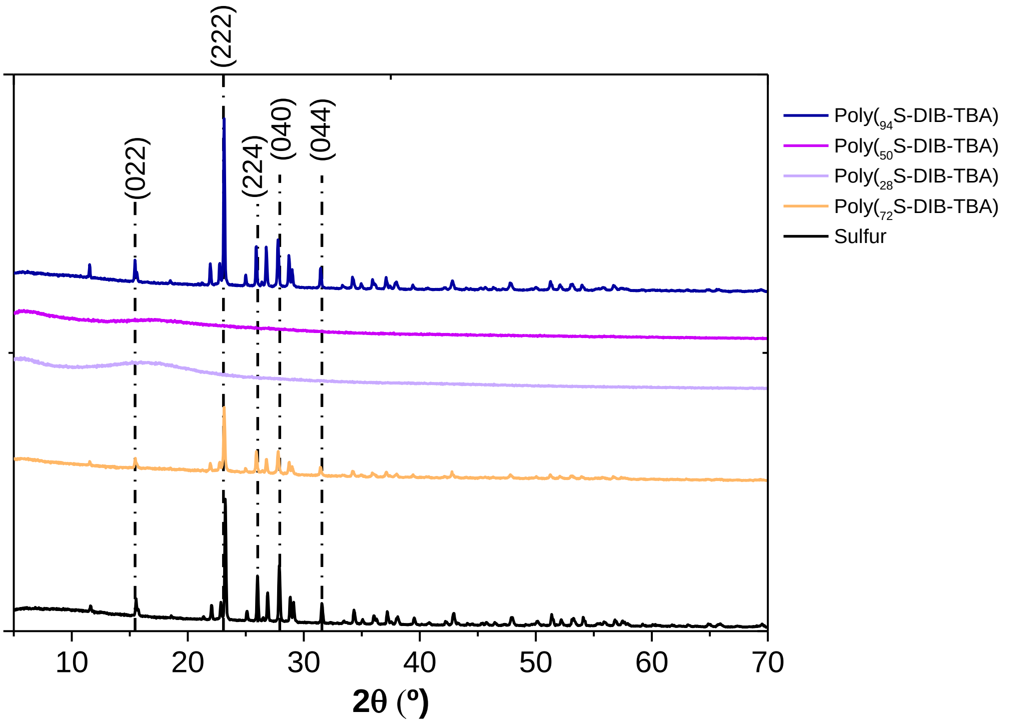

Figure S8: PXRD spectra of the poly(S-DIB-TBA) series.

S2.4 DSC

The temperature at which monoclinic sulfur melts is around 119 °C, which is considered as the melting point of elemental sulfur. This transition was observed in the poly(S_{72}-DIB-TBA) (Figure S8), which is consistent with the PXRD data. However, despite the presence of unreacted sulfur indicated by PXRD analysis in the poly(S_{94}-DIB-TBA), no such indication was observed by DSC curves. This could be due to the lower sensitivity of DSC compared to PXRD analysis, which might not have detected the unreacted sulfur in the polymer. Moreover, the glass transition temperature (T_g) decreased significantly with higher sulfur content in the polymer. For instance, the poly(S_{28}-DIB-TBA) exhibited the highest T_g of 22.9 °C.

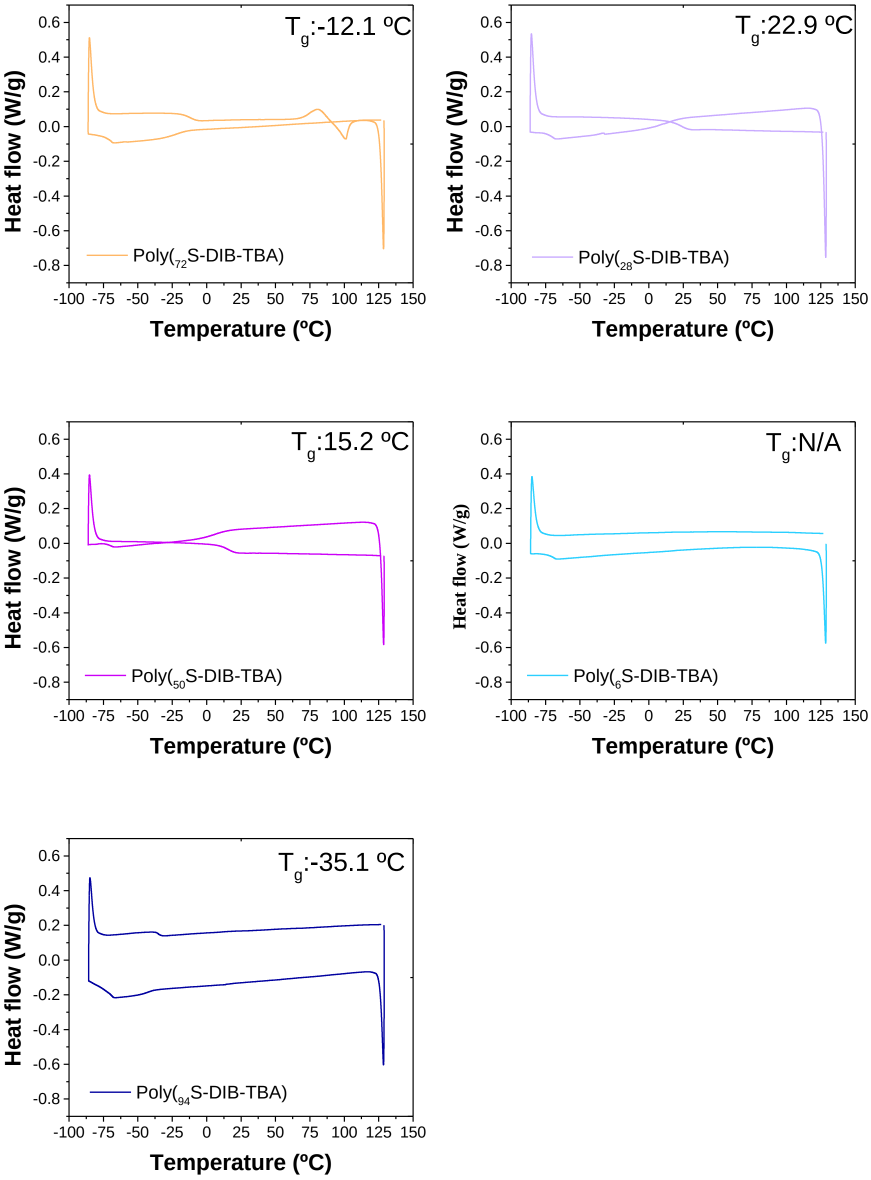

Figure S9: DSC thermograms of the poly(S-DIB-TBA) series.

S3. Atomic force microscopy (AFM) of the coating

The following AFM data was collected from regions of the sample that lacked microscale roughness. Therefore, the AFM images appear smooth compared to the SEM images. In addition, the nanosized surface features were made up of fumed silica particles (Ø ~ 10-20 nm), which is similar to that of the AFM probe tip (SCM-PIT-V2, Ø – 20 nm). However, the significant surface roughness is clearly demonstrated by complementary SEM,

WCA, and RA data, confirming substantial micro- and nanoscale texture. Furthermore, statistical analysis (Table S3) highlights the superhydrophobic properties as a synergistic result of the surface roughness and hydrophobic surface chemistry.

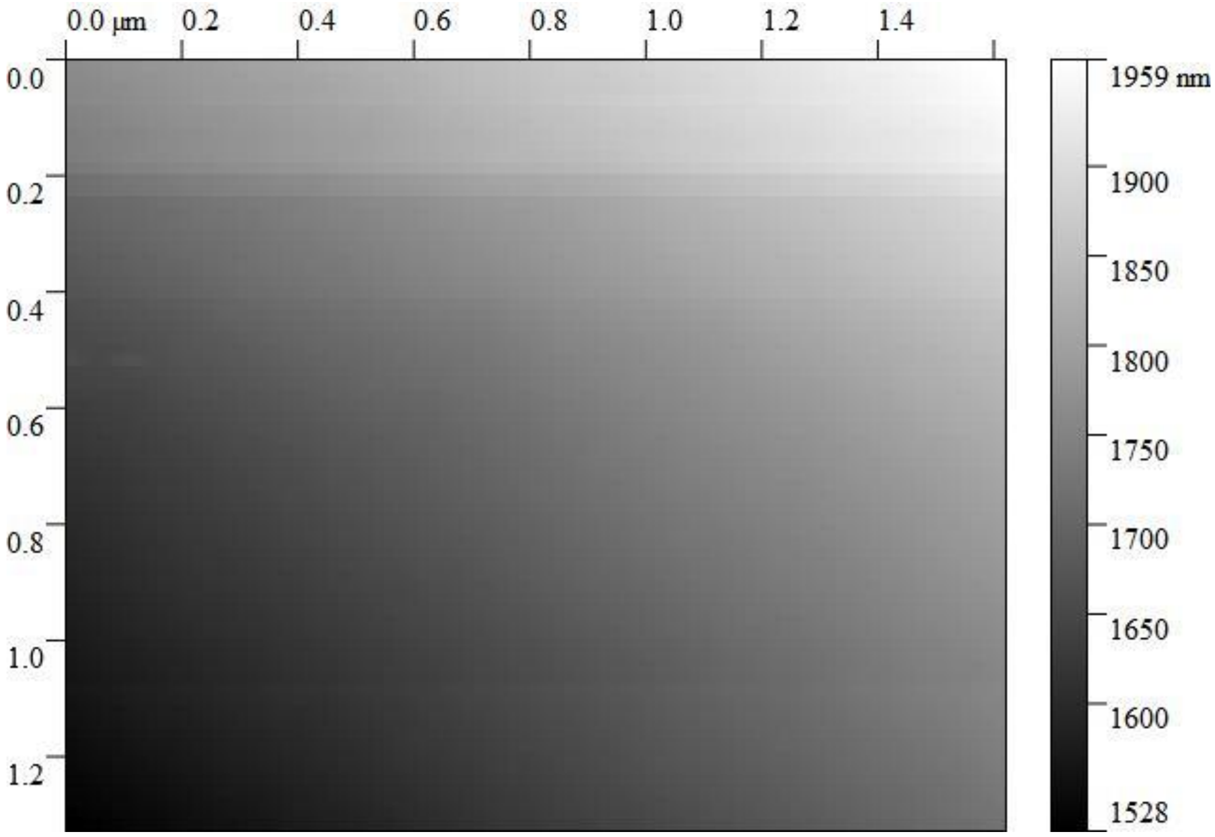

Figure S10: AFM image of the coating 17 (see Table S2).

Table S3: Statistical parameters of the AFM measurement.

**Parameter**	**Result**
RMS roughness	89.77 nm
Mean roughness	73.29 nm
Skew	0.2204
Surface area	2.22 μm^2

S4. Poly(S-DIB) and poly(S-DIB-Zn(DTC)$_2$) characterization.

S4.1 FTIR

As observed for the poly(S-DIB-TBA) series (Figure S1), there was a consumption of the -CH_2 in the DIB molecule and the generation of C-S bonds in the poly(S_{28}-DIB) and poly(S_{28}-DIB-Zn(DTC)$_2$) structure, which can be confirmed by the disappearance of the 886 cm^{-1} band and the appearance of the 695 cm^{-1} band, respectively (Figure S9).

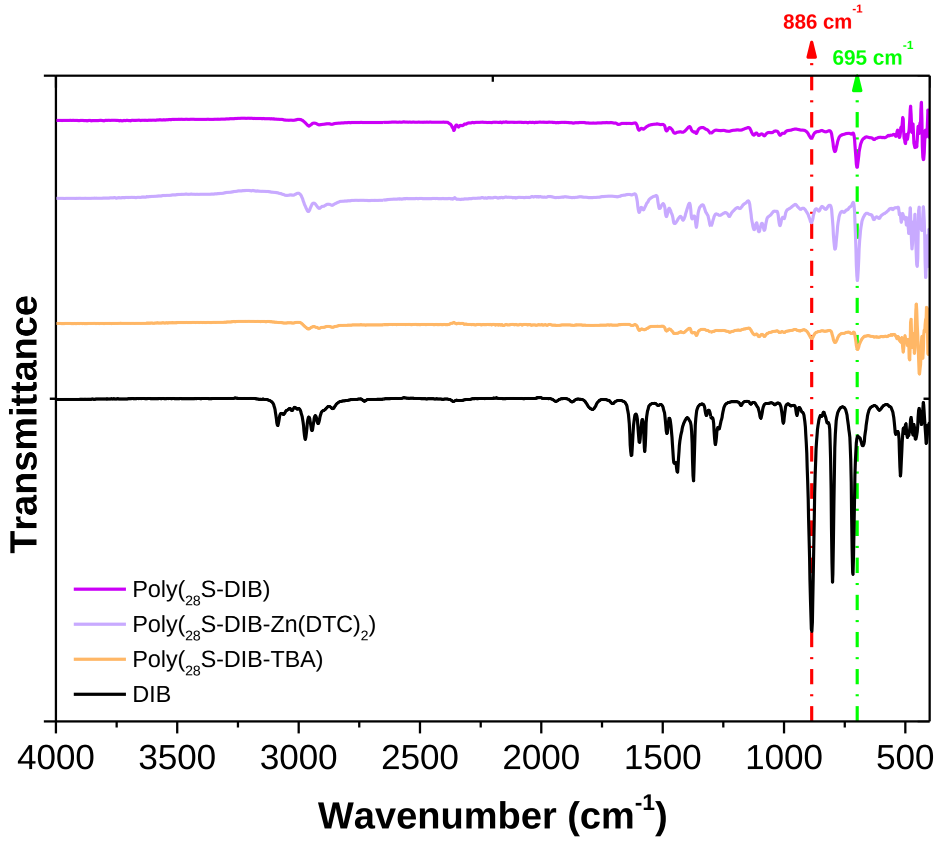

Figure S11: FTIR spectra of the poly(S_{28}-DIB), poly(S_{28}-DIB-TBA), and poly(S_{28}-DIB-Zn(DTC)$_2$).

S4.2 NMR

As observed in the poly(S-DIB-TBA) series, both poly($_{28}$S-DIB)and poly($_{28}$S-DIB-Zn(DTC)$_2$) contained methyl protons groups ($\delta = 1.0$–2.2 ppm) and methylene groups in the polymer backbone ($\delta = 2.9$–3.4 ppm) (Figure S10 and S11). Additionally, no unreacted methylene proton groups were observed ($\delta = 5.10$–5.40 ppm).

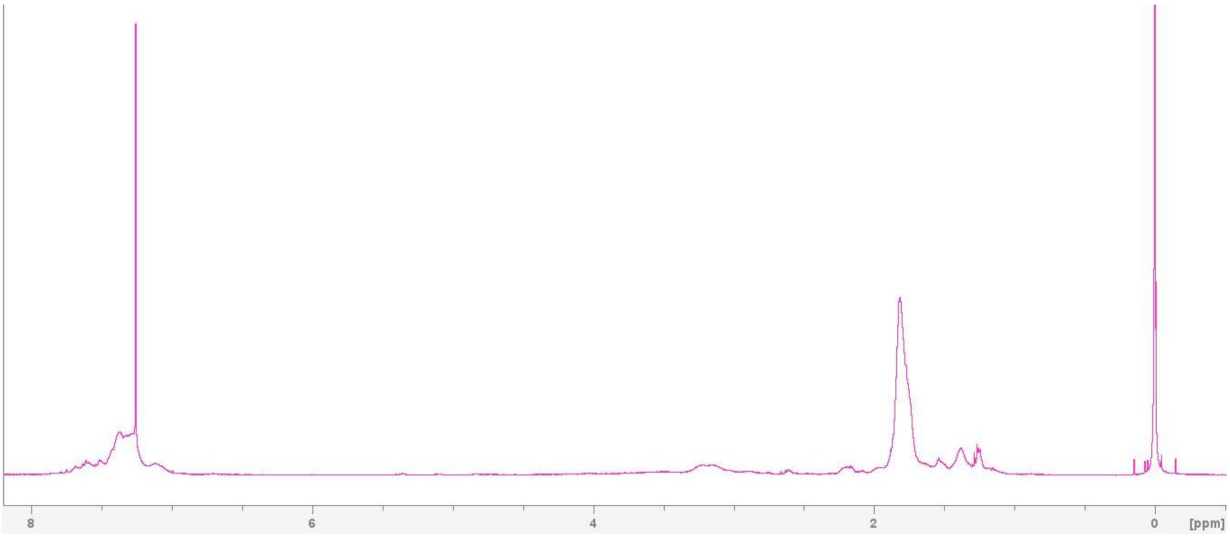

Figure S12: ^{1}H-NMR spectrum of poly($_{28}$S-DIB-Zn(DTC)$_2$).

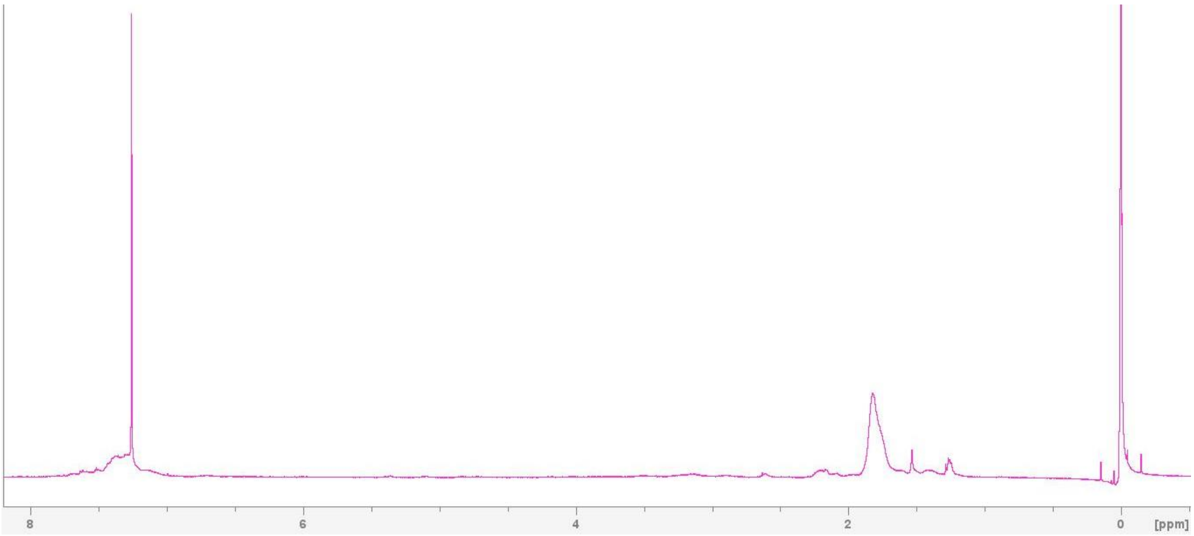

Figure S13: ^{1}H-NMR spectrum of poly($_{28}$S-DIB).

S4.3 PXRD

As observed in Figure S7, characteristic diffraction peaks of sulfur appeared at 2θ = 16º 23°, 27°, 28°, and 32° (Figure S7). However, they were not observed in the spectra of poly($_{28}$S-DIB-TBA), poly($_{28}$S-DIB), and poly($_{28}$S-DIB-Zn(DTC)$_2$), indicating a total consumption of crystalline sulfur during the IV copolymerization (Figure S12).

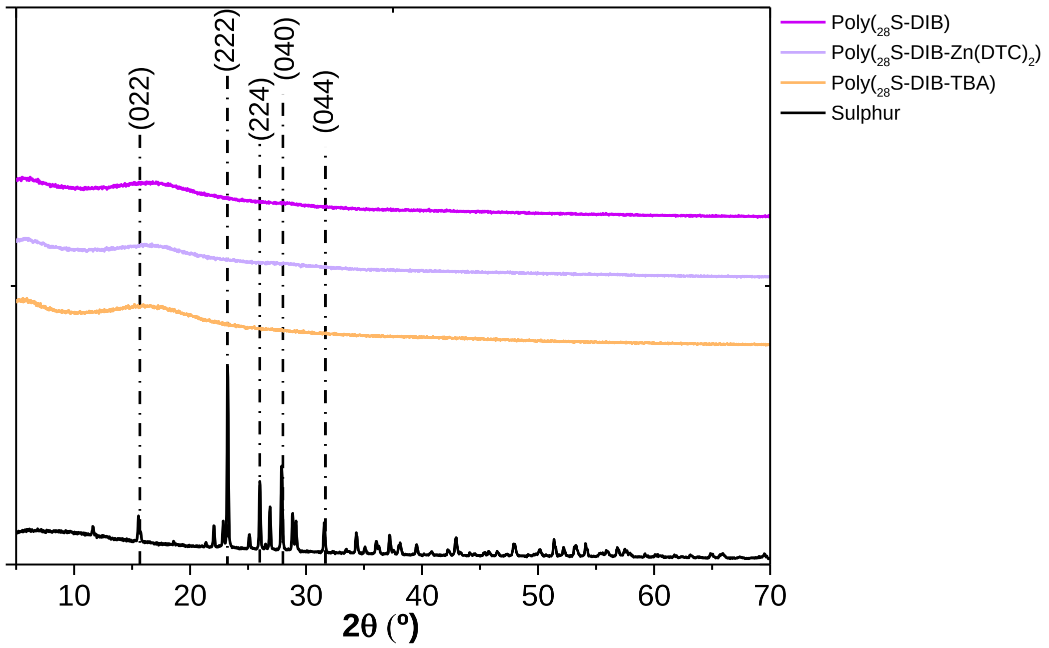

Figure S14: PXRD spectra of the poly(S_{28}-DIB), poly(S_{28}-DIB-TBA), and poly(S_{28}-DIB-Zn(DTC)$_2$).

S.4.4 DSC

As observed for poly(S_{28}-DIB-TBA) (Figure S8), no residual sulfur was detected in the poly(S_{28}-DIB-TBA) and poly(S_{28}-DIB-Zn(DTC)$_2$) (Figure S13), which corroborates with the PXRD data (Figure S12). Additionally, when compared to poly(S_{28}-DIB-TBA), both poly(S_{28}-DIB-TBA) and poly(S_{28}-DIB-Zn(DTC)$_2$) showed higher T_g with the latter showing the highest one.

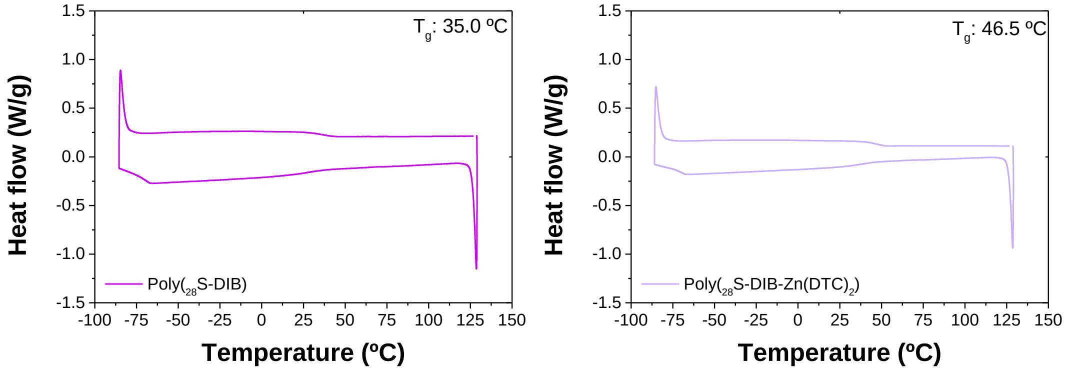

Figure S15: DSC thermograms of poly(S_{28}-DIB-TBA) and poly(S_{28}-DIB-Zn(DTC)$_2$).

.

S.4.5 TGA

Thermogravimetric analysis of poly(S_{28}-DIB), poly(S_{28}-DIB-TBA), and poly(S_{28}-DIB-Zn(DTC)$_2$) revealed similar mass loss pattern among the polymers but with different extension within temperate range between 50 – 600 °C (Figure S14). For instance, poly(S_{28}-DIB) showed the lowest residual content at 600 °C of 16.5%; meanwhile, poly(S_{28}-DIB-TBA) and poly(S_{28}-DIB-Zn(DTC)$_2$) showed a residual content of 22.0% and 29.8%. Additionally, the degradation pattern observed suggests the formation of short chains of cross-linked oligomers for all the polymers and longer-chain of polysulfides.

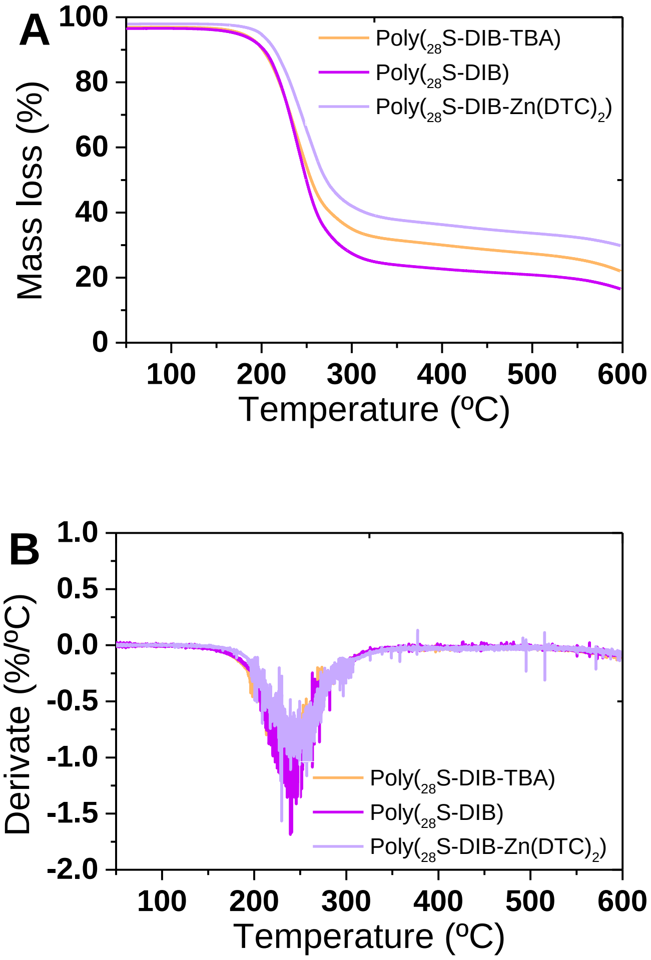

Figure S16: TGA (A) and DTG (B) curve of poly(S_{28}-DIB), poly(S_{28}-DIB-TBA), and poly(S_{28}-DIB-Zn(DTC)$_2$).

S5. Experimental

S5.1 Materials

1,3-diisopropenybenzene (DIB) (stabilized with TBC, ≥9.0%) Elemental sulfur (S_8, powder, ≥99.0%), toluene, and tributylamine (TBA) (≥9.0%) were purchased from Tokyo Chemical Industry (UK). Carbon nanofibers (CNFs) (graphitized (iron-free) 100 nm x 20 – 200 µm, hexamethyldisilazane (≥99.0), SiO_2 (10-20 nm), tetramethylsilane (analytical standard for NMR), and zinc(II) diethyldithiocarbamate ($Zn(DTC)_2$) (97.0%) were purchased from Merck Ltd. (UK). Chloroform (99.8%) were purchased from Fischer Scientific Ltd. (UK). Deuterated chloroform (99.8%) was purchased from Cambridge Isotopes Laboratories Ltd. (UK).

S5.2 Characterization of poly(S-DIB)

Scanning Electron Microscopy (SEM) imaging and Energy-Dispersive X-Ray spectroscopy (EDS) were performed using an FEI Inspect F system with an operational acceleration voltage of 10–20 kV. To enhance electrical conductivity within the SEM, samples were sputter-coated with a thin layer of gold using an Automatic Sputter Coater. Fourier Transform Infrared (FTIR) spectra were recorded using a Bruker Tensor 27 instrument over the wavenumber range of 500 to 4000 cm^{-1}. To analyze surface topography, atomic force microscopy (AFM) was used. AFM of the samples were analyzed by Bruker Dimension Icon. Nuclear Magnetic Resonance (NMR) analysis utilized a Bruker Advance DRX (400 MHz) spectrometer, with deuterated chloroform as the solvent and tetramethylsilane as the internal standard. Differential Scanning Calorimetry (DSC) measurements were carried out using a TA Instruments Discovery Series DSC 25. A heat-cool-heat method was employed, with heating and cooling rates set at 10 °C min^{-1} under nitrogen atmosphere, spanning from -60 to 150 °C. Powder X-Ray diffraction (PXRD) patterns were collected in reflection mode using a Panalytical X'Pert PRO MPD equipped with a high throughput screening XYZ stage, X-ray focusing mirror, and PIXcel detector. Cu Kα radiation (λ = 1.5406 Å) was utilized, and data were collected over a range of 5–70° (step ≈ 0.03°) using loose powder samples on thin Mylar film within aluminum well plates. Thermogravimetric analysis (TGA) was conducted under an inert atmosphere on a TA Instruments TGA 5500. Heating was carried out at a heating rate of 10 ºC min^{-1}, from room temperature to 600 °C.

S5.3 Characterization of coatings

The Ossila contact angle V3.0.0.0 software was used to determine WCA using 10 µL water droplets (n = 4). UV stability was evaluated indirectly by monitoring changes in the

WCA over a given exposure period. The coatings were positioned 6.8 cm below the Cole-Parmer Handheld UV Lamp λ ~ 254 nm at a power of 6 W. Rolling angles were determined by tilting the surface and noting the angle at which the water droplet-initiated rolling. A minimum of four tilting angle measurements were conducted and then averaged for each reported value to ensure accuracy. The mechanical stability of the coating was assessed using tape peeling tests. This involved applying office tape (Scotch® 508, 3M) to the coating and rolling a 250 g weight across it before peeling off the tape. The change in WCA was subsequently measured in four distinct areas to provide a qualitative stability characterization. The same procedure was followed to determine mechanical stability using Rotary Platform Taber 1700 Single Head Rotary Abraser. The test involved the rotation of two abrading wheels (CS-0, Calibrase) coated with S-42 sandpaper strips in the opposite direction compared to the sample rotation with a 100 g load. The thermal stability of the coatings was assessed using a heating chamber with forced convection (Binder, UK). The coatings were heated for 1 h before the WCA measurements for each temperature. All the statistical analyses were conducted using Statistica® 7.1.
